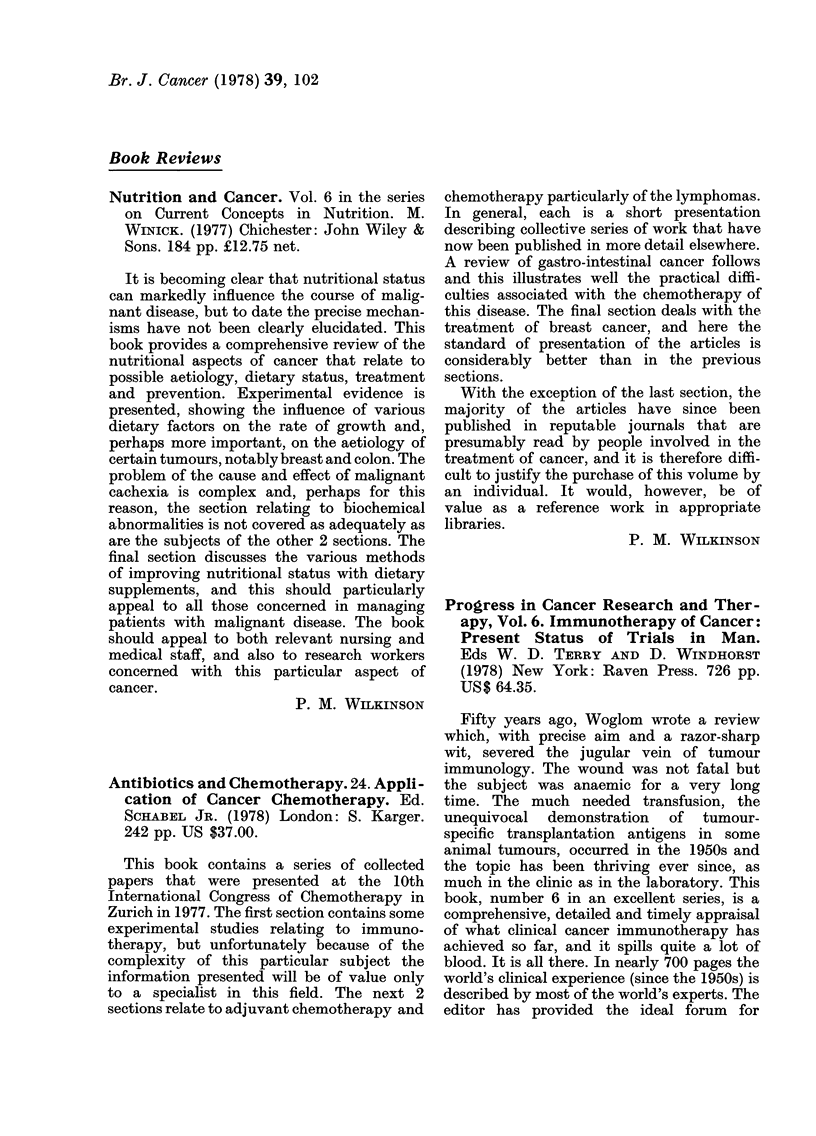# Antibiotics and Chemotherapy. 24. Application of Cancer Chemotherapy

**Published:** 1979-01

**Authors:** P. M. Wilkinson


P. M. WILKINSON

**Antibiotics and Chemotherapy. 24. Application of Cancer Chemotherapy.** Ed. SCHABEL JR. (1978) London: S. Karger. 242 pp. US $37.00.

This book contains a series of collected papers that were presented at the 10th International Congress of Chemotherapy in Zurich in 1977. The first section contains some experimental studies relating to immunotherapy, but unfortunately because of the complexity of this particular subject the information presented will be of value only to a specialist in this field. The next 2 sections relate to adjuvant chemotherapy and chemotherapy particularly of the lymphomas. In general, each is a short presentation describing collective series of work that have now been published in more detail elsewhere. A review of gastro-intestinal cancer follows and this illustrates well the practical difficulties associated with the chemotherapy of this disease. The final section deals with the treatment of breast cancer, and here the standard of presentation of the articles is considerably better than in the previous sections.

With the exception of the last section, the majority of the articles have since been published in reputable journals that are presumably read by people involved in the treatment of cancer, and it is therefore difficult to justify the purchase of this volume by an individual. It would, however, be of value as a reference work in appropriate libraries.